# Outcomes of Initial Transcorporal Versus Standard Placement of Artificial Urinary Sphincter in Patients With Prior Radiation

**DOI:** 10.7759/cureus.25519

**Published:** 2022-05-31

**Authors:** David Miller, Kelly Pekala, Xueying Zhang, Oluwaseun Orikogbo, Devin Rogers, Thomas W Fuller, Avinash Maganty, Paul Rusilko

**Affiliations:** 1 Department of Urology, University of Pittsburgh Medical Center, Pittsburgh, USA; 2 Department of Statistics, University of Pittsburgh, Pittsburgh, USA; 3 Department of Urology, University of Pittsburgh, Pittsburgh, USA; 4 Department of Urology, Virginia Mason Medical Center, Seattle, USA

**Keywords:** artificial urinary sphincter, urinary incontinence, urethra/radiation effects, quality of life, prostatectomy/adverse effects

## Abstract

Objective: This study aimed to evaluate both device and functional outcomes of men who underwent initial artificial urinary sphincter (AUS) placement after pelvic radiation using the transcorporal versus the standard approach.

Methods: A retrospective review of patients who underwent first-time AUS placement after pelvic irradiation for prostate cancer was conducted between January 2008 and June 2020. Patients were grouped by transcorporal versus standard device placement. The primary outcomes of interest included major complications (revision or explant surgery) and functional outcomes (pads per day, International Prostate Symptom Score {IPSS}, quality of life {QOL} score).

Results: We identified 45 patients who underwent first-time AUS with a history of prior pelvic irradiation for prostate cancer, 27 underwent transcorporal placement and 18 underwent standard placement. Transcorporal AUS placement resulted in a significantly lower number of major complications (p=0.01), explants (p=0.02), and revisions (p=0.04) The transcorporal artificial urinary sphincter group had better postoperative pads per day (p=0.04), IPSS (p<0.01), and IPSS QOL score (p<0.01).

Conclusions: Initial transcorporal artificial urinary sphincter placement is a promising technique with lower rates of major complications in patients with a history of prior pelvic radiation and had better functional urinary outcomes.

## Introduction

The artificial urethral sphincter is the gold standard treatment for male stress urinary incontinence which commonly results from prostatectomy or pelvic radiation for prostate cancer. Radiation therapy is a well-established risk factor for complications following artificial urinary sphincter placement including an increased risk of sphincter erosion and need for revision surgery [1,2]. This increased risk of erosion is thought to be due to a compromise to urethral blood supply caused by radiation-induced obliterative endarteritis [3-5]. This mechanism parallels several other identified risk factors including hypertension, cardiovascular disease, prior urethroplasty, and prior sphincter erosion in which the urethral blood supply is also compromised [3,6-8]. 

Despite significant risks associated with artificial urinary sphincter, specifically the need for future revision or device replacement, patient satisfaction rates remain high [9]. Several techniques are available to mitigate the risks of erosion in high-risk patients such as urethral wrapping with a xenograft or transcorporal artificial urinary sphincter placement. Such techniques provide an additional tissue barrier between the thinner dorsal corpora spongiosum and urethra. The transcorporal method specifically interposes ventral corporal cavernosa tunica albuginea between the cuff and urethra, and thereby theoretically reduces the risk of erosion [10]. However, the degree to which this technique reduces the future risk of erosion and its impact on functional outcomes remains an area of ongoing debate. One recent study by Redmond et. al. showed trends toward lower rates of erosion and need for revisions with the transcorporal approach [11]. However, a large study by Ortiz et. al. did not demonstrate the protective effect of transcorporal placement compared with standard sphincter placement [12].

Our objective was to study the impact on complications and functional outcomes of initial artificial urinary sphincter placement via the transcorporal approach. To our knowledge, this is one of the first studies evaluating initial transcorporal artificial urinary sphincter (AUS) placement versus the standard approach. We hypothesized that men with prostate cancer and prior pelvic irradiation who underwent initial placement of an artificial urinary sphincter via a transcorporal approach would have improved outcomes compared to men who underwent standard placement. The initial results of this study were previously presented at the Sexual Medicine Society of North America (SMSNA) annual meeting on November 14, 2020 [13].

## Materials and methods

Study population

After institutional review board approval (STUDY19080339), we conducted a retrospective review of patients who underwent first-time artificial urinary sphincter placement between January 2008 and June 2020 after pelvic irradiation as part of their treatment for prostate cancer. Patients with a history of pelvic radiation for other reasons (e.g., rectal cancer) were excluded. Patients undergoing anything other than initial AUS surgery (e.g., dual cuffs) and men who underwent radiation after AUS placement were excluded. The decision to proceed with either transcorporal or standard AUS placement was based on history, risk factors, erectile function, plans for additional penile prosthesis surgery with shared decision making between surgeon and patient.

Surgical techniques

The artificial urinary sphincters (AMS 800^TM^; Minnetonka, MN: American Medical Systems) were implanted by two high-volume implant surgeons who are facile with both the standard and transcorporal techniques both of which used the traditional perineal approach. Patients were grouped by surgical approach for analysis.

Data collection and analysis

We collected patient demographic characteristics including age, body mass index (BMI), tobacco use, cardiovascular disease, diabetes mellitus, prior urethral dilation, urethrotomy or transurethral resection for bladder neck contracture, prior urethroplasty, prior urethral sling, prior bulking agent, radiation type (external beam radiotherapy, intensity-modulated radiation therapy, low dose rate brachytherapy, high dose rate brachytherapy), timing of radiation (salvage, adjuvant), radiation dose, timing of radiation to AUS implant, and American Society of Anesthesiologists (ASA) class. We collected device characteristics including cuff size and cuff pressure. Major complications and reasons for additional surgery were subdivided into mechanical, urethral atrophy, and infection/erosion. Functional outcomes including International Prostate Symptom Score (IPSS), quality of life (QOL), and pads per day were recorded preoperatively and postoperatively. Length of follow-up from date of surgery to last follow-up appointment was calculated.

Primary and secondary​​​​​ outcomes of interest

Our primary outcome of interest was occurrence of a major complication, defined as the need for revision or explant surgery, following AUS implant. We recorded the reasons for revision or explant surgery and categorized them as infection/erosion, mechanical device malfunction, or urethral atrophy.​​​​​​​ Our secondary outcomes of interest were patient functional outcomes measured by pads per day, IPSS with QOL score and need for urinary diversion after AUS placement.

Statistical analysis

All analyses were performed using RStudio (version 1.2.5025; Boston, MA: RStudio, Inc.) and a p-value of 0.05 was set for statistical significance. We used t-tests and Wilcoxon tests to check the differences in demographic characteristics based on the distribution assumption. Wilcoxon tests were used to compare the postoperative pads per day and IPSS scores between the transcorporal and standard approaches. To describe the probability of developing complication, we obtained Cox proportional hazard function over time with age, BMI, and cardiovascular disease and used the likelihood ratio test to compare the risk. Kaplan-Meier curve was used to visually show the device survival probability.

## Results

Demographics

A total of 45 patients underwent first-time artificial urinary sphincter with a history of prior pelvic irradiation for prostate cancer. There were 27 patients who underwent transcorporal placement with a mean follow-up of 32 months and 18 patients who underwent standard placement with a mean follow-up of 39 months. There were no significant differences in the cohort with regards to age, BMI, tobacco use, androgen deprivation therapy, ASA class, except for cardiovascular disease (p=0.026). The difference in radiation dosage was significant between the groups with standard AUS receiving 69.8 Gy and the transcorporal group receiving 67 Gy (p=0.05). The majority of patients received salvage radiation. The median time from the last dose of radiation to artificial urinary sphincter implant was 86 months. Additionally, the standard group had a high percentage of history of prior urethral surgery 61% as compared to the transcorporal group 31% which was significant (p=0.05). The median size of AUS cuff was 4.5 cm (range: 3.5-5.5cm) for the transcorporal group and 4.0 cm (range: 3.5-4.5cm) for the standard group which was a significant difference (p=0.05). A pressure regulating balloon with a standard cuff pressure of 61-70 cm H_2_O was used in all except two cases which were done via the standard approach (Table 1).

**Table 1:** Patient characteristics: demographic information including comorbidities, radiation dosage received, history of prior urethral procedures ASA: American Society of Anesthesiologists; TUR: transurethral

Characteristic		Standard AUS N=18 (%)	Transcorporal AUS N=27 (%)	p-Value
Mean age ±SD (years)		70.6±6.5	73.7 ±7.2	0.15
Median BMI (kg/m^2^)		30.5	28.89	0.46
Median ASA score (range)		3 (2-4)	3 (2-3)	0.91
Tobacco use		12	13	0.36
Androgen deprivation therapy		4	13	0.12
Cardiovascular disease		15 (83)	15 (56)	0.026
Diabetes mellitus		7 (39)	7 (26)	0.24
Prior urethral surgery (dilation, urethrotomy, or TUR for anastomotic contracture)		11 (61)	9 (33)	0.05
Bulking agent		0 (0)	2 (7)	0.27
Sling		1 (6)	3 (1)	0.58
Urethroplasty		0 (0)	2 (7)	0.27
Median radiation dose (Gy) (range)		69.79 (66.6-78.0)	67.0 (66.6-68.4)	0.05
Type of radiation	Adjuvant	2 (11)	5 (19)	0.68
	Salvage	16 (89)	20 (74)	0.28
	Primary	0 (0)	2 (7)	-
Median time from radiation to device implant months (range)		62 (13-170)	108 (8-288)	0.23
Median length of follow-up months (range)		39 (2-135)	32 (2-100)	0.45
Cuff-size (cm)		4.0 (3.5-4.5)	4.5 (3.5-5.5)	0.05
Cuff-pressure (cm H_2_O)		61-70	61-70	1

Primary outcome (major complication)

The major complication rate defined as revision or explant surgery was 56% in the standard group compared to 14.8% in the transcorporal group (p<0.01). Transcorporal AUS placement resulted in a significantly lower number of explants (p=0.02) with only a 7.4% explant rate in this group as opposed to 39% in the standard group. All explants were done for either infection or erosion. The revision rate was 33% in the standard group and 7.4% in the transcorporal group (p=0.04) (Table 2).

**Table 2:** Patient complications by surgical technique AUS: artificial urinary sphincter

	Standard AUS N (%)	Transcorporal AUS N (%)	p-Value
Major complication: either AUS revision or explant	10 (56)	4 (14.8)	0.01
Median time to complication (AUS revision or explant) months	46	19	0.01
AUS explant	7 (39)	2 (7)	0.02
AUS revision	6 (33)	2 (7)	0.04

**Figure 1:**
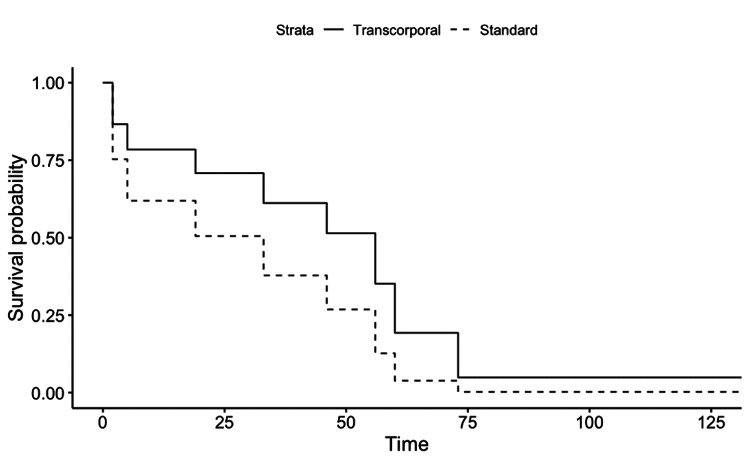
Artificial urinary sphincter device complication free probability Kaplan-Meier curve showing time (months) to device complication (revision or explantation) probability for transcorporal AUS compared with standard AUS placement. AUS: artificial urinary sphincter

All AUS revisions were for either urethral atrophy or mechanical reasons, none were done for erosion. Cox proportional hazard function showed no significant device survival differences between the two groups (p=0.24). However, as seen on the Kaplan-Meier curves the transcorporal group trended toward having a lower risk for AUS complications (Figure 1).

Secondary outcome (patient functional outcomes)

Both transcorporal and standard approaches resulted in improvements in pads per day and IPSS after artificial urinary sphincter implantation, based on the Mann-Whitney test results. However, the transcorporal AUS group had superior outcomes in terms of postoperative pads per day (p=0.04), postoperative IPSS (p<0.01), and postoperative IPSS QOL score (p<0.01) (Table 3).

**Table 3:** Patient reported functional outcomes AUS: artificial urinary sphincter; IPSS: International Prostate Symptom Score; QOL: quality of life

	Standard AUS (N=18)	Transcorporal AUS (N=27)	p-Value
Median pads per day last follow up (range)	3 (0-7) (N=12)	1 (0-7) (N=27)	0.019
Median IPSS postop (range)	16 (8-24) (N=7)	5 (1-15) (N=24)	0.001
IPSS postop QOL (range)	3 (1-6) (N=8)	1 (0-5) (N=24)	0.01

## Discussion

We report that transcorporal AUS placement resulted in a significantly lower number of major complications, explants, and revisions. Additionally, our study is important in that it captures a relatively long follow-up period of a median of 32 months for transcorporal AUS patients and 39 months for standard AUS patients. A recent large study reported that the median time to AUS explanation in irradiated patients is 26.4 months, thus we should be capturing the majority of potential complications within our cohort [2]. 

The increased risk of sphincter erosion and need for revision for patients with history of pelvic radiation has been well established [2,10-15]. A large meta-analysis found that 37% of men with a history of pelvic irradiation underwent surgical revision compared to 20% for non-irradiated men. Furthermore, the rates of surgical revision did not improve over a 25-year time period (1989-2014) of the study affirming the need for innovation [1]. Irradiated men may benefit from transcorporal placement of AUS, where a thick fibrous buttress between the cuff and urethra is created at the time of initial AUS placement. However, retrospective data does not show superiority of a transcorporal approach in cases of reimplantation after erosion with or without prior radiation. In these studies, the transcorporal approach was used for re-operative cases and revision surgery following sphincter erosion instead of first-time sphincter placement [2,10-15]. 

Functional outcomes after AUS placement in men with a history of radiation remain controversial in the literature. Some studies report equally high satisfaction rates of ~90% in men with and without a history of pelvic radiation despite an increase in complications such as need for revision surgery and equivalent functional outcomes in terms of incontinence, yet others report persistent urinary incontinence at rates of ~40% [15-18]. In this study, we utilized pads per day use to measure the degree of incontinence as this measure has been previously shown to correlate well with the degree of incontinence [19]. There was a statistically significant improvement in pad per day use after surgery regardless of surgical approach. The transcorporal AUS group had better outcomes in terms of postoperative pads per day, postoperative IPSS, and postoperative IPSS QoL score. These findings may be due to the much lower rate of explantation in the transcorporal group compared to those in the standard group resulting in more patients having an AUS on the last follow-up.

The most commonly cited pitfall of the transcorporal approach is violation of the tunica albuginea of the corporal bodies, which can result in new erectile dysfunction [7,8]. This complication has likely impacted the adoption of the transcorporal approach, particularly in the initial sphincter placement setting. However, successful penile prosthesis placement after transcorporal artificial urinary sphincter is well described [20]. Notably, none of the patients who underwent transcorporal AUS placement in our cohort have elected to undergo penile prosthesis placement despite preoperative counseling that this was an option for them should they wish to pursue it. The risk of new erectile dysfunction can be ameliorated with the newly described Gullwing modification to the transcorporal approach. In this technique, rectangular-shaped flaps are raised from the ventral corpora and wrapped circumferentially around the urethra for increased urethral buttressing. These flap defects are then repaired with xenograft (e.g., bovine pericardium) or allograft (e.g., cadaveric dermis) which prevents narrowing of the corporal bodies [21,22]. 

Our results must be interpreted in the context of several limitations. First, the small sample size may impact the statistical power to adequately assess for outcome differences. Second, the decision to proceed with either transcorporal or standard artificial urinary sphincter placement was made via shared decision-making. Thus, patients were not randomized which introduces a possible selection bias, although we found patient characteristics to be similar for both groups. Third, functional outcomes were assessed with pads per day in lieu of a standardized questionnaire. Fourth, due to the relatively recent adoption of transcorporal technique at our institution, we are not able to report on late complications. However, follow-up was greater than two years for both groups. Finally, our study was retrospective in nature and thus susceptible to the biases associated with such studies. 

## Conclusions

Initial transcorporal AUS placement is a promising technique with lower rates of major complications with a significantly lower number of explants due to erosion or infection compared with standard placement. Additionally, transcorporal AUS patients had better functional urinary outcomes with improved postoperative IPSS and QOL score as well as decreased number of pads per day compared to patients who underwent the standard approach. Initial transcorporal placement of AUS should be considered in patients who have fragile urethras due to history of pelvic radiation.
